# Potential of lipid metabolism in marine diatoms for biofuel production

**DOI:** 10.1186/s13068-015-0212-4

**Published:** 2015-02-22

**Authors:** Giuliana d’Ippolito, Angela Sardo, Debora Paris, Filomena Monica Vella, Maria Grazia Adelfi, Pierpaolo Botte, Carmela Gallo, Angelo Fontana

**Affiliations:** Istituto di Chimica Biomolecolare (ICB) - CNR, Via Campi Flegrei 34, 80078 Pozzuoli, NA Italy

**Keywords:** Diatoms, Biofuels, Lipid metabolism, Microalgae, Bioenergy

## Abstract

**Background:**

Diatoms are an ecologically relevant group of microalgae that are not commonly considered for bio-oil production even if they are responsible for massive blooms at sea. Seventeen diatom species were screened for their capacity to produce biomass and lipids, in relation to their growth rate. Triglyceride levels were also assessed as a preferential source of biofuels.

**Results:**

Using statistical analysis, two centric diatoms, *Thalassiosira weissflogii* and *Cyclotella cryptica*, were selected as good candidates for oil production. Lipid levels significantly increased when the two diatoms were cultivated in a two-stage process under nitrogen limitation. The effect was less pronounced in cultures where silicon was reduced to 20% of the standard supply. Nitrogen limitation did not affect growth rates but led to lipid remodeling and *de novo* synthesis of triacylglycerols.

**Conclusions:**

Triacylglycerols in *T. weissflogii* and *C. cryptica* can account for up to 82% and 88% of total glycerolipids, thereby suggesting that the two species are promising candidates for large-scale experimentation for biofuel production.

**Electronic supplementary material:**

The online version of this article (doi:10.1186/s13068-015-0212-4) contains supplementary material, which is available to authorized users.

## Background

Diatoms (Bacillariophyta) are photosynthetic unicellular organisms with characteristic silica cell walls (frustules). Over 8,000 different species are described growing worldwide in lakes and at sea, but according to different authors [1-3], extant species are estimated to range between 20,000 and 200,000 species. The lineage is traditionally divided into two orders: centric diatoms or *Centrales* that are radially symmetrical and pennate diatoms or *Pennales* that are bilaterally symmetrical. The first order is further subdivided into polar and non-polar centrics, whereas the latter order includes the classes Bacillariophyceae and Fragilariophyceae according to the presence or absence of a raphe [4]. Although usually described as microalgae, diatoms have very distinctive traits that set them apart from other photoautotrophic eukaryotes [5-8].

These microorganisms are the main component of the phytoplankton and play a major role in the global cycling of carbon and silicon at sea [9,10]. In particular, their photosynthetic activity contributes to almost half of oceanic primary productivity. As such, diatoms are the major component of the so-called biological carbon pump [11], and the burial of diatoms over geologic time has produced an important fraction of petroleum deposits [12].

Diatoms produce oil drops that are stored intracellularly as a reserve material during the vegetative period of growth, with percentages that vary from less than 23% to greater than 45% of dry cell weight [13]. Physiological and genetic manipulations have also showed the possibility of increasing the amount of lipids in the cellular mass and re-invigorated studies regarding the potential of affording oil production by these microorganisms [14].

The present work is focused on the potential of diatoms as a source of biofuels, especially planktonic species that are responsible for massive algal blooms in the ocean. The aims of this study were (1) to test biomass and lipid productivity of 17 diatom species comparing these results with those obtained from four non-diatom microalgae chosen among the genera traditionally considered for oil production [15-17]; (2) to select promising diatom species by applying principal component analysis (PCA); and (3) to verify the response to silicon (Si) and nitrogen (N) limitation on lipid metabolism by a two-stage cultivation model [18].

## Results and discussion

### Cell growth and principal component analysis on chemical and biochemical parameters

Established cultures of 17 diatom species and four species of green microalgae belonging to the classes Eustigmatophyceae and Chlorophyceae were grown in standard f/2 medium. Each microalgal strain showed specific slope and duration of the growth curves (Additional file 1: Figures S1 and S2), which presumably also reflected variability in cellular and metabolic responses. Since it was not possible to determine a homogenous and common day of harvesting for each strain, each culture was stopped in the stationary phase, when the slope of the growth curve showed a negative ratio of the vertical change to the horizontal change between two consecutive cell counts. According to the physiology of each species, this transition occurred within time intervals ranging from a few days to a few weeks.

Main cultivation parameters and gross chemical production of each microalgal strain are summarized in Table 1. Diatom cells were generally larger than those of non-diatom species (Additional file 1: Table S1), showing lower cell density but higher growth rates, as measured by the number of cellular divisions (doubling time, *T*_*d*_). The fastest growing strains (*Chaetoceros* species, *Thalassiosira rotula*, *Thalassiosira weissflogii* P09, *Cylindrotheca fusiformis*, and *Pseudo*-*nitzschia pseudodelicatissima*) doubled three times faster than green microalgae (15 to 17 h doubling time in diatoms compared to 44-83 h doubling time in non-diatoms). Despite the difference in absolute biomass production (that is, 700 mg L^−1^ for *Nannochloropsis salina*; 500 mg L^−1^ for *Dunaliella tertiolecta*; 195 mg L^−1^ of *C. fusiformis*; 146 mg L^−1^*T. weissflogii* P09; 247 mg L^−1^ of *Phaeodactylum tricornutum*), the productivity (mg L^−1^ day^−1^) of diatom species was comparable to non-diatoms (for example, 33 mg L^−1^ day^−1^*N. salina*; 36 mg L^−1^ day^−1^*D. tertiolecta*; 27 mg L^−1^ day^−1^*C. fusiformis*; 24 mg L^−1^ day^−1^*T. weissflogii* P09; and *P. tricornutum* 22 mg L^−1^ day^−1^). On the other hand, lipids in a few diatom species (for example, *T. weissflogii* CCMP 1010 or *Cyclotella cryptica* CCMP 331) accounted for almost 40% of the total organic matter, whereas the other microalgae, including the non-diatoms, had lipid levels usually below 30% of the total biomass. A few diatoms also showed a very high content of triacylglycerols (TAG) that comprised more than 50% of the lipid content.

**Table 1:** **Chemical and biochemical parameters analyzed in cultures of 17 marine diatoms compared to other marine microalgae**

**Class**	**Microalae**	**Onset stationary phase (days)**	**Duplication time (h)**	**Biomass productivity (mg L** ^**−1**^ **day** ^**−1**^ **)**	**Lipid productivity (mg L** ^**−1**^ **day** ^**−1**^ **)**	**Percentage of lipids**	**Percentage of FFA**	**Percentage of TAG**	**Percentage of GL**	**Percentage of PL**
Bacillariophyceae	*Chaetoceros curvisetus* CCMP 3260	7	18	13.22 ± 0.66	1.97 ± 0.04	14.86 ± 0.31	100	―	―	―
	*Chaetoceros socialis* CCMP 3263	7	16	2.47 ± 0.07	0.19 ± 0.01	7.63 ± 0.23	100	―	―	―
	*Chaetoceros affinis* CCMP 3259	7	17	4.13 ± 0.17	0.50 ± 0.01	12.11 ± 0.30	100	―	―	―
	*Thalassiosira rotula* CCMP 1647	7	17	5.44 ± 0.33	0.43 ± 0.01	7.95 ± 0.26	100	―	―	―
	*Thalassiosira rotula* CCMP 3264	7	19	4.35 ± 0.22	0.54 ± 0.02	12.42 ± 0.37	100	―	―	―
	*Thalassiosira weissflogii* P09	6	16	24.29 ± 0.97	7.27 ± 0.28	29.94 ± 1.17	3.0 ± 0.2	51.0 ± 3.2	30.0 ± 1.0	16.0 ± 1.0
	*Thalassiosira weissflogii* CCMP 1010	6	22	12.53 ± 0.44	4.87 ± 0.10	38.84 ± 0.78	8.0 ± 0.3	53.0 ± 1.9	21.0 ± 1.3	18.0 ± 1.1
	*Thalassiosira weissflogii* CCMP 1336	6	24	14.84 ± 0.45	3.48 ± 0.10	23.48 ± 0.66	5.0 ± 0.3	45.0 ± 3.2	31.0 ± 1.3	19.0 ± 1.1
	*Thalassiosira pseudonana* CCMP 1335	8	24	5.87 ± 0.24	1.72 ± 0.07	29.33 ± 1.17	5.0 ± 0.3	19.0 ± 0.9	55.0 ± 1.8	21.0 ± 1.0
	*Cyclotella cryptica* CCMP 331	13	48	7.11 ± 0.14	2.98 ± 0.09	41.97 ± 1.26	0	55.0 ± 2.1	24.0 ± 1.7	21.0 ± 1.0
	*Skeletonema marinoi* CCMP 2092	7	23	9.02 ± 0.54	0.85 ± 0.02	9.38 ± 0.23	100	―	―	―
	*Skeletonema marinoi* CCMP 2052	7	16	7.17 ± 0.27	0.66 ± 0.03	9.14 ± 0.43	100	―	―	―
	*Cylindrotheca fusiformis* CCMP 343	7	15	27.27 ± 1.09	4.78 ± 0.18	17.51 ± 0.67	7.0 ± 0.2	18.0 ± 0.7	54.0 ± 3.2	21.0 ± 0.5
	*Phaeodactylum tricornutum* CCMP 632	11	29	22.44 ± 0.90	2.09 ± 0.06	9.32 ± 0.28	11.0 ± 0.7	19.0 ± 0.6	44.0 ± 2.1	26.0 ± 1.0
	*Pseudo-nitzschia pseudodelicatissima* B317	10	15	4.12 ± 0.11	0.56 ± 0.03	13.50 ± 0.81	100	―	―	―
	*Ditylum brightwelli* CCMP 358	12	34	4.66 ± 0.28	0.57 ± 0.03	12.20 ± 0.71	52.0 ± 3.6	―	19.0 ± 0.8	29.0 ± 1.8
	*Melosira octogona* CCMP 483	10	25	9.10 ± 0.44	1.88 ± 0.11	20.60 ± 1.24	2.0 ± 0.1	29.0 ± 1.8	38.0 ± 1.2	31.0 ± 1.9
Eustigmatophyceae	*Nannochloropsis salina* CCMP 369	21	44	33.09 ± 1.16	7.27 ± 0.31	21.98 ± 0.92	14.0 ± 0.5	20.0 ± 0.5	39.0 ± 2.3	27.0 ± 1.6
Chlorophyceae	*Dunaliella salina* CCAP19/18	20	83	19.79 ± 0.99	5.22 ± 0.17	26.36 ± 0.87	6.0 ± 0.3	7.0 ± 0.3	49.0 ± 2.1	38.0 ± 2.3
	*Dunaliella tertiolecta* CCMP 1320	14	51	36.42 ± 1.02	9.71 ± 0.44	26.65 ± 1.20	5.0 ± 0.2	17.0 ± 1.2	53.0 ± 1.7	25.0 ± 1.1
	*Chlamydomonas* sp. CCMP 222	12	30	14.26 ± 0.53	2.98 ± 0.15	20.92 ± 1.05	3.0 ± 0.2	7.0 ± 0.3	60.0 ± 3.7	30.0 ± 1.0

Duplication time, biomass and lipid productivity (mg L^−1^ day^−1^), percentage of lipids (percentage of dry weight), percentage of FFA, and percentage of TAG (percentage of total lipid extract) were used for PCA analysis. FFA, free fatty acid; GL, glycolipid; PL, phospholipid; TAG, triacylglycerol.

**Figure 1:**
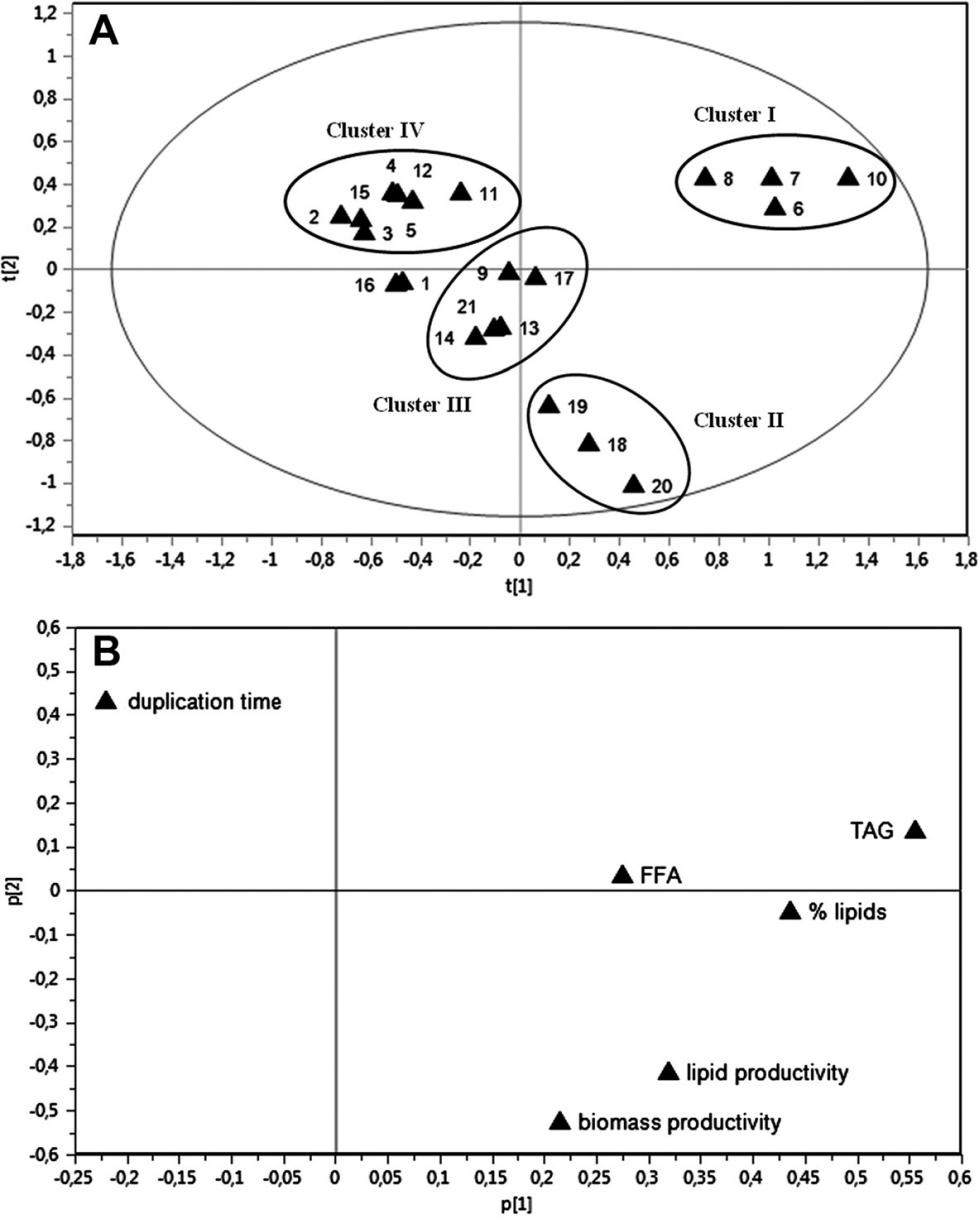
**PCA analysis for species distribution in two components statistical model. (A)** Scores plot and **(B)** loadings plot with the parameters responsible for the clusterization. 1 Chaetoceros curvisetus; 2 Chaetoceros socialis; 3 Chaetoceros affinis; 4 Thalassiosira rotula 1647; 5 Thalassiosira rotula 3264; 6 Thalassiosira weissflogii P09; 7 Thalassiosira weissflogii 1010; 8 Thalassiosira weissflogii 1336; 9 Thalassiosira pseudonana; 10 Cyclotella cryptica; 11 Skeletonema marinoi 2092; 12 Skeletonema marinoi 2052; 13 Cylindrotheca fusiformis; 14 Phaeodactylum tricornutum; 15 Pseudo-nitzschia pseudodelicatissima; 16 Ditylum brightwelli; 17 Melosira octogona; 18 Nannochloropsis salina; 19 Dunaliella salina; 20 Dunaliella tertiolecta; 21 Chlamydomonas sp.

Data were processed by PCA which modeled and described the variation of biomass and chemical descriptors (productivity of biomass and lipids) together with quantitative loadings on the composition of the lipid fraction (percentage of lipids per cell dry weight and percentage of triglycerides per total lipid content), stability of the lipid components (free fatty acids content), and culture rate (duplication time). All variables were normalized by a range scaling method and reciprocal values were used for duplication time and free fatty acids content, which negatively affected the selection process. With these constraints, replicates of microalgal species clustered in four well-defined groups with robust statistical significance (about 80%) and an overall clear separation of diatom from non-diatom samples (Figure 1A). The associated loading plot highlighted the contribution of each parameter to this distribution (Figure 1B).

Four species of centric diatoms of the order Thalassiosirales, namely *C. cryptica* and strains of *T. weissflogii* (cluster I), grouped in a restricted area of the first quadrant of the plot. Major difference in duplication time and lipid percentage, especially triacylglycerols, determined the separation of these species from *D. salina*, *D. tertiolecta*, and *N. salina* (cluster II) although lipid productivity in the two groups was in some cases comparable (for example, 7.3 mg L^−1^ day^−1^ for *T. weissflogii P09* and *N. salina* or 9.7 mg L^−1^ day^−1^ for *D. tertiolecta*). Cluster II was characterized by high productivity and included the algal genera that have attracted considerable interest in the search for biological candidates for production of energy from biomass [15,19]. Of the green microalgae, only *Chlamydomonas* sp. was found in another cluster together with *T. pseudonana*, *C. fusiformis*, *P. tricornutum*, and *Melosira octogona* (cluster III) as result of the fairly small level of TAG in comparison to clusters I and II. It is interesting to note that cluster III included *T. pseudonana* and *P. tricornutum* that are the most studied diatom species for production of biofuels up to now [20-22]. The remaining microalgae were distributed in the fourth quadrant of the plot. They comprised most of the species (cluster IV) with very short duplication time (between 15 and 23 h). In comparison to cluster I, these diatoms showed low productivity and strong hydrolytic enzymatic activity that was responsible for releasing fatty acids from complex lipids [23,24]. This enzymatic process caused a decrease in the major lipid classes and a consequent increase in the levels of free fatty acids that negatively affected the biotechnological importance of these species as potential sources of biofuel.

### Biomass and lipid productivity of *T. weissflogii* P09 and *C. cryptica* under nutrient limitation

The effect of nutrient limitation on algal metabolism has been reported as a powerful tool to increase oil productivity. The practical use of nutrient limitation to induce TAG production involves growing cells under nutrient replete conditions to high biomass, followed by limitation of nutrient supply. The amount of lipid production that can be induced is theoretically enormous and justifies the technical hurdles of this approach from an industrial viewpoint [15,25]. To date, the amount of lipid production under nutrient stress for diatoms has sometimes been controversial. Reduction of silicon and nitrogen in cultures of the diatoms *P. tricornutum* and *T. pseudonana* is reported to increase lipid production, especially triglycerides, but this result is often counterbalanced by a reduction in growth rate [20,26,27] suggesting that generalizations on the effects of nutrient limitation may not be universal. Traller and Hildebrand have shown changes of TAG accumulation rate over time and sub-population variability of TAG production in *C. cryptica*, which is probably challenging to overcome in a bulk system [28]. Recently, Li and co-workers showed that nitrogen limitation under low light increased oil levels in *P. tricornutum* without affecting culture growth [18]. The authors used a two-stage cultivation strategy which consisted in a nutritionally replete initial phase of growth followed by a lipid induction phase under nutrient-limited conditions, as also recorded for other microalgae [15,25]. In compliance with this work, two diatoms of cluster I, namely *T. weissflogii* P09 and *C. cryptica*, were cultured under low light (200 μE) and harvested in the stationary phase by gentle centrifugation. The pellet was split, and cells were transferred in replete or limited media. These two strains showed different characteristics that allowed comparison of two distinct models of diatom culture production. In fact, *T. weissflogii* P09 was characterized by high biomass and lipid productivity that were comparable to those of the best strains of cluster II (Table 1). On the other hand, *C. cryptica* showed the highest percentage of lipids (almost 42% of the organic extract) and TAG (55% of the lipid pool) of the 21 strains considered in this study. Since silicon deficiency arrests cell cycle progression at the G1/S or G2/M transition for most diatoms [29], a limited f/2 medium containing silica and nitrogen reduced to 20% of their normal supply was used. The secondary cultures were maintained at 200 μE until cells entered the stationary phase once again.

**Figure 2:**
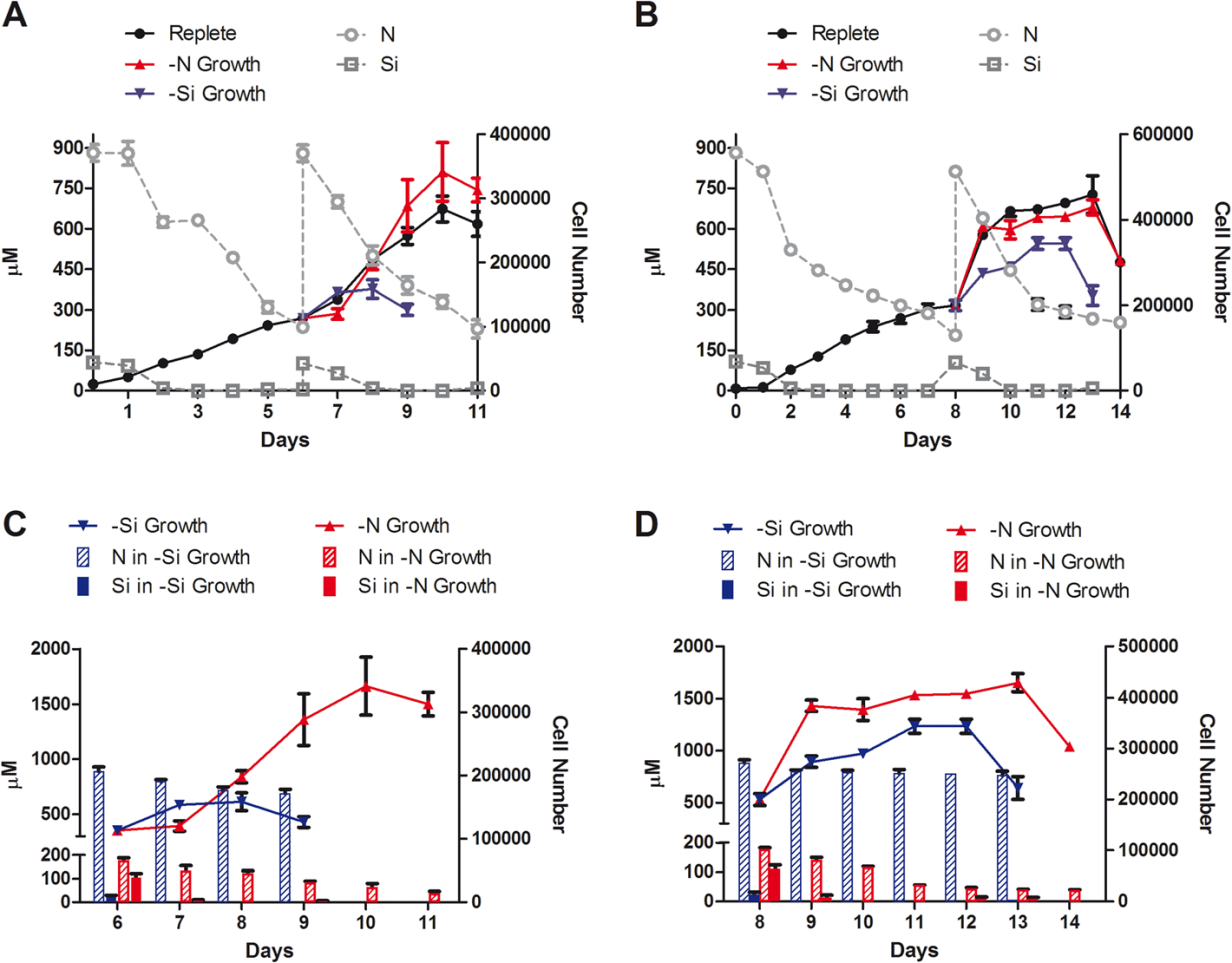
**Cultures of T. weissflogii P09 and C. cryptica CCMP 331 under two-stage nutrient regime. (A)** Growth curves during the first (from day 0 to day 6) and second (from day 6 to day 11) stage of growth for Thalassiosira weissflogii . Gray lines indicate nutrient consumption under replete conditions; **(B)** growth curves during the first (from day 0 to day 8) and second (from day 8 to day 14) stage of growth for C. cryptica. Gray lines indicate nutrient consumption under replete conditions; **(C)** growth curve and nutrient consumption under depleted conditions (second stage of growth) for Thalassiosira weissflogii P09; **(D)** growth curve and nutrient consumption under depleted conditions (second stage of growth) for C. cryptica.

As shown in Figure 2, the two diatoms consumed both silicon and nitrogen at different extent during the exponential growth phase. In agreement with the literature [30-32], after 2 days, silicon had diminished to close to zero, whereas nitrogen levels had decreased more slowly but were nonetheless reduced to one third of the starting supply by the time cells had entered the stationary phase (6 days for *T. weissflogii* P09 and 8 days for *C. cryptica*) (Figure 2A,B). A similar response was also recorded during the second stage of growth when cultures were gently centrifuged and re-suspended in both replete and nutrient-limited media. Since silicon is an essential nutrient for diatoms [33], it was immediately incorporated when added to the medium, independently of the culture conditions of both species (Figure 2C,D). On the other hand, nitrogen was consumed to a small extent in Si-deprived cultures. Interestingly, the two species responded differently to Si limitation, since *C. cryptica* grew better than *T. weissflogii* P09 when Si was reduced or absent in the medium.

**Figure 3:**
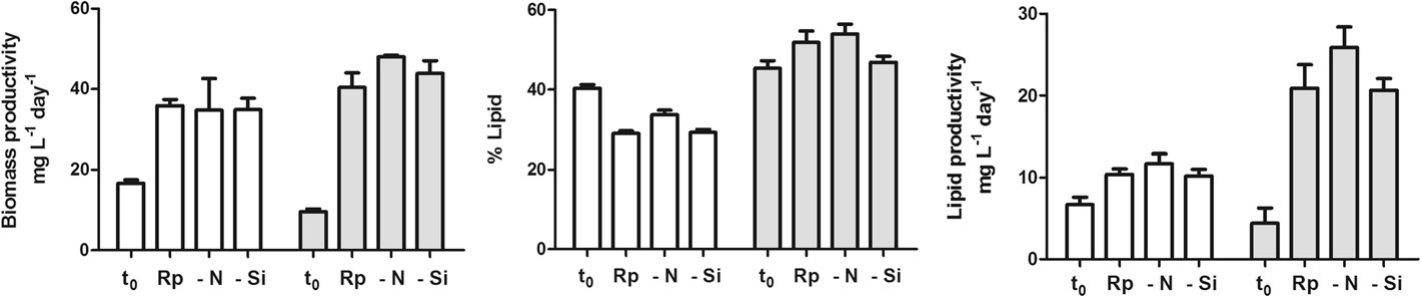
**Effect of nutrient depletion on biomass and lipid production in the centric diatoms T. weissoflogii (white) and C. cryptica (gray).** t_0_ = starting point of limitation experiments; Rp = replete conditions; −N = nitrogen limitation (20% of standard concentration in f/2 medium); −Si = silicon limitation (20% of standard concentration in f/2 medium).

Simple refreshment with nutrient-replete media (Rp culture) strongly boosted biomass productivity that doubled in the case of *T. weissflogii* and quadrupled in *C. cryptica* (Figure 3). On the other hand, replacement with both replete and depleted-nutrient media induced a general reduction in the percentage of total lipids in the biomass of *T. weissflogii* even if absolute productivity increased due to biomass accumulation. In comparison with Rp conditions, nitrogen and silicon limitation did not cause significant changes in growth probably because cultures were already partially nutrient limited when they entered into the stationary phase. Under the tested growth conditions, only *C. cryptica* showed a slight increase in biomass in N-limited conditions but this effect did not result in a similar increase in lipids. Surprisingly, these results were not consistent with previous reports that showed a boost in lipid production of diatoms when silicon was limited compared to nitrogen limitation [14]. In fact, silicon is not directly coupled to cellular metabolism of diatoms [34,35], but its depletion leads to arrest of cell division and consequent accumulation of organic carbon, mostly in the form of TAG [36,37]. Nevertheless, the response is not general. To the best of our knowledge, the effects of nutrient modulation on lipid metabolism in *T. weissflogii* have been never reported even if heterogeneity in neutral lipid accumulation over time and within individual cells of *C. cryptica* under silicon or nitrogen limitation has been recently described [14,28]. In line with this study, Jeffryes and co-workers have also underlined the importance of tuning silicon delivery in order to improve lipid productivity and to maintain a basal concentration that is necessary to maintain physiological activity of the cells [30].

### Lipid analysis of *T. weissflogii* and *C. cryptica* under nutrient limitation

**Figure 4:**
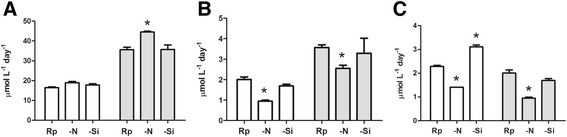
**Variation of (A) triacylglycerols (B) glycolipids and (C) phospholipids in T. weissflogii (white) and C. cryptica (gray).** Rp = replete conditions; −N = nitrogen limitation; −Si = silicon limitation. *p < 0.05.

Although both polar and neutral lipids can be converted to biodiesel [38,39], TAG represent the ideal fraction since they are easily trans-esterified by alkaline methanol. Diatoms accumulate TAG as a reserve material during the vegetative growth period, but little is known regarding their metabolism and relationship with other lipid classes [12,40]. Recently, a new nuclear magnetic resonance (NMR)-based analytical protocol was introduced for rapid quantitative estimation of TAG and other lipid pools in underivatized extracts of microalgae [41]. Proton NMR spectra of the extracts of *T. weissflogii* and *C. cryptica* (Additional file 1: Figures S3 and S4) show that TAG were the main glycerolipid class (above 70%). Figure 4 reports productivity (μmol L^−1^ day^−1^) of TAG, glycoglycerolipids (GL), and phospholipids (PL) in these species under different nutrient regimes. In replete conditions, TAG showed level (16.5 and 35.6 μmol L^−1^ day^−1^, respectively) that were almost ten times higher than GL (2.0 and 3.6 μmol L^−1^ day^−1^) and up to twenty times more than PL (2.3 and 1.7 μmol L^−1^ day^−1^). In agreement with data on nutrient consumption shown in Figure 2, these results suggest that TAG hyper-accumulate under the conditions used in this study, in agreement with those by Traller and Hildebrand [28]. Growth of both diatoms in silica limitation did not induce changes in TAG levels, whereas nitrogen limitation induced an additional increase (almost 20%) in TAG productivity (19 μmol L^−1^ day^−1^ in *T. weissflogii* and 45 μmol L^−1^ day^−1^ in *C. cryptica*) in comparison with replete conditions. This process occurred to the detriment of GL and PL in both species even if *C. cryptica* showed an increase in molar productivity of TAG that was significantly higher than the sum of the diminution of the other two lipid classes. In agreement with recent reports for *P. tricornutum* [18,42], these data seem to indicate that nitrogen limitation stimulates TAG accumulation by both *de novo* synthesis and remodeling of membrane glycerolipids. In particular, the former pathway is largely dominant in *C. cryptica*, whereas remodeling of membrane lipids, especially GL, is the main mechanism of oil synthesis in *T. weissflogii*. In this process, the remodeled lipids show a sort of molecular signature by maintaining the fatty acid profiles of the original lipid classes [43,44]. Under the tested growth conditions, fatty acid composition of *T. weissflogii* and *C. cryptica* revealed a substantial increase of eicosapentaenoic acid in TAG under nitrogen limitation (Additional file 1: Table S2), which was in agreement with the results on molar productivity.

## Conclusions

In this study, 17 different strains of marine diatoms were analyzed for biomass productivity and chemical composition. Statistical methods based on multivariate analysis selected two strains of *T. weissflogii* and *C. cryptica* as best candidates for biofuel production. Under the tested growth conditions, these diatoms showed lipid productivity that were comparable to green microalgae of the genera, for example, *Nannochloropsis* and *Dunaliella*, commonly proposed as potential sources of biofuels. Both diatom candidates were responsive to two-stage cultivation strategies that caused an increase in biomass and lipid productivity. Under nitrogen limitation, a remarkably high level of TAG (above 80% of glycerolipids) production occurred as a result of remodeling of the lipid pools and *de novo* synthesis of neutral lipids, thereby suggesting that appropriate changes in the culture conditions can be effective tools to modulate lipid metabolism and positively affect oil production in these photoautotrophic organisms.

Although further large-scale studies are necessary to fully evaluate the productivity of these species, the present study highlights the plasticity of the lipid pools and the phenotypic accumulation of TAG suggesting that other metabolic studies and more tools are necessary to exploit large-scale culture of diatoms as sources of biofuels. The metabolic response of diatom cells to different culture conditions is possibly dependent not only on nutrient limitation but also on intrinsic predisposition of the cells related to different growth phases and metabolic stages. Presumably, these factors are more important in outdoor production systems that in more controlled lab cultures.

## Methods

### Microorganisms and culture conditions

All strains were obtained from the National Center for Marine Algae and Microbiota (Bigelow Laboratory for Ocean Sciences, USA), except for *T. weissflogii* P09 and *Pseudo-nitzschia pseudodelicatissima* (local strains, isolated from the Gulf of Naples, Italy), and *D. salina* CCAP 19/18 (Culture Collection of Algae and Protozoa, Oban, Scotland). These included diatoms (*Chaetoceros curvisetus* CCMP 3260, *Chaetoceros socialis* CCMP 3263, *Chaetoceros affinis* CCMP 3259, *T. rotula* CCMP 1647, *T. rotula* CCMP 3264, *T. weissflogii* CCMP 1010, *T. weissflogii* CCMP 1336, *T. pseudonana* CCMP 1335, *C. cryptica* CCMP 331, *Skeletonema marinoi* CCMP 2092, *S. marinoi* CCMP 2052, *Ditylum brightwelli* CCMP 358, *M. octogona* CCMP 483, *C. fusiformis* CCMP 343, and *P. tricornutum* CCMP 632) and green microalgae of the classes Eustigmatophyceae (*N. salina* CCMP 369) and Chlorophyceae (*D. tertiolecta* CCMP 1320 and *Chlamydomonas* sp*.* CCMP 222). The inocula were all taken from healthy exponentially growing cultures. Cultures of each strain were carried out in triplicate using a 10% volume of algal inocula. Microalgae were cultured in triplicates in 2-L polycarbonate flasks in f/2 medium at 20°C and gently bubbled with sterile air [45]. Artificial light intensity (200 μmol m^−2^ s^−1^) was provided by daylight fluorescent tube with a 14:10 h light:dark photoperiod. Cell growth (cells mL^−1^) was estimated daily using a Bürker counting chamber (Merck, Leuven, Belgium) (depth 0.100 mm) under an inverted microscope (Nikon Eclipse TE200, Nikon Corp., Tokyo, Japan). Cell division was expressed as doubling time (*T*_*d*_), calculated according to Equation 1, where *N*_1_ and *N*_2_ are cell numbers at time 1 (*t*_1_) and time 2 (*t*_2_) at the extremes of the linear phase, according to Wood *et al*. [46]:

1 $$ {T}_d=\left({t}_2-{t}_1\right)\times 1\mathrm{n}2/\left(1\mathrm{n}\;{N}_2/{N}_1\right). $$

Microalgae were harvested when the slope of the growth curve was very small or negative compared to the exponential phase by centrifugation in a swing-out Allegra X12R (Beckman Coulter Inc., Palo Alto, CA, USA) at 2,300 g for 10 (Bacillariophyceae) or 15 min (Eustigmatophyceae and Chlorophyceae). Cells were washed twice with ammonium formate 0.5 M to remove salt [16] and immediately frozen in liquid nitrogen. The pellets were then lyophilized with a MicroModulyo 230 (Thermo Electron Corporation, Milford, MA, USA) freeze dryer, to estimate cell dry weight. Biomass productivity was expressed as dry weight from 1 L of culture per days of algal growth.

### Chemical analysis

Unless otherwise specified, lipid extraction was performed using the modified Folch method [47]. Lipid content (mg L^−1^) was determined gravimetrically by weighting lipid extracts. Lipid composition (percentage of free fatty acids, triglycerides, glycolipids, and phospholipids) was estimated after purification on silica gel column of each class of molecules. Lipid extracts were methylated with diazomethane and purified by adsorption chromatography on silica gel in accordance to ref. [23]. Total fatty acid composition was determined by GC-MS on the corresponding methyl esters (fatty acid methyl ester (FAME)) obtained after saponification of lipid extracts with Na_2_CO_3_ in MeOH (42°C) for 4 h. FAME mixtures were analyzed with a gas chromatograph equipped with a mass spectrometer (Focus GC-PolarisQ, Thermo Fisher Scientific, Waltham, MA, USA) (injector 260°C; detector 260°C; temperature gradient 160°C up to 260°C, 5°C/min). Pentadecanoic acid was used as internal standard. Protein content was determined by Lowry method (RC DC protein assay; Bio-Rad) on 20 mL of culture. Carbohydrate content was determined using the phenol-sulfuric acid method [48] on 100 mL of culture.

### Nutrient limitation experiments

Diatom cultures were initially maintained in optimal conditions for cell growth in 10-L polycarbonate carboys containing 6.5 L of culture. The cultures were harvested after the onset of the stationary phase (6 days for *T. weissflogii* and 8 days for *C. cryptica*) and centrifuged as reported above. The supernatant was discarded, and the cell pellet was washed twice with nitrate- and silicate-free f/2 medium. The culture was then divided into three aliquots, and cells were collected by centrifugation. The resulting pellets were re-inoculated into 2-L fresh f/2 medium containing 882 μmol L^−1^ of nitrate and 106 μmol L^−1^ silicate (replete, Rp), 177 μmol L^−1^ of nitrate, and 106 μmol L^−1^ silicate (Nitrate-limited media, −N) and 882 μmol L^−1^ of nitrate and 20 μmol L^−1^ silicate (Silicate-limited media, −Si). Each experiment was run in triplicate. Algal suspensions were collected in the beginning of the declining phase and treated as described above. Biomass and lipid productivity under nutrient limitation were determined in accordance with the formula:

$$ {\mathrm{Productivity}}_i=\frac{C_i\;\left({t}_{\mathrm{end}}\right)-{C}_i\;\left({t}_0\right)}{t_{\mathrm{end}}-{t}_0} $$

where *C*_*i*_ is the concentration of the component of interest *i*, (in which *i* represents either biomass dry weight, lipid extract, or μmol of glycerolipids [40], and *t* time (days). Quantification of lipids (μmoles of triglycerides, glycolipids, and phospholipids) was assessed by NMR on DRX600 (Bruker, Germany) equipped with Cryoprobe and operating at 600 MHz, in accordance to a recent published NMR methodology [41]. During the experiments, 10 mL of culture was centrifuged daily and the supernatant was filtered at 0.22 μm for nutrient analysis. Dissolved nitrate and silicate were estimated with the spectrophotometric method according to references [49,50].

### Principal component analysis and statistical treatment of the data

Biomass productivity (mg L^−1^ day^−1^), lipid productivity (mg L^−1^ day^−1^), duplication time, percentage of total lipids, percentage of free fatty acids, and percentage of neutral lipids were used for statistical analysis. These biological parameters were treated with a range scaling method [51,52]. Reciprocal values were used for duplication time and free fatty acids whose minimal values positively affected cultures. Multivariate statistical analysis was performed with SIMCA-P+ 12 package (Umetrics, Umea, Sweden). ANOVA test was used for statistical analysis of measured parameters with Bonferroni correction.

## Additional file

Additional file 1:
**Supporting material.** Figure S1. Growth curves of non-diatom species that were considered in this study. See Experimental section for culture conditions. Figure S2. Growth curves of non-diatom species that were considered in this study. See Experimental section for culture conditions. Figure S3. Proton NMR spectra of extracts of *T. weissflogii* under Si-limited (a), N-limited (b), and replete (c) conditions. Figure S4. Proton NMR spectra of extracts of *C. cryptica* under Si-limited (a), N-limited (b), and replete (c) conditions. Table S1. Cell size of microalgae considered in this study. Table S2. Fatty acid composition of TAG in *T. weissflogii* and *C. cryptica* under nutrient limitation.

## Abbreviations

FAME: Fatty acid methyl ester
GL: Glycoglycerolipid
GC-MS: Gas chromatography-mass spectrometry
NMR: Nuclear magnetic resonance
PCA: Principal component analysis
PL: Phospholipid
TAG: Triacylglycerol
