# Supplementary material for: Potential of lipid metabolism in marine diatoms for biofuel production
Source: Biotechnol Biofuels. 2015 Feb 22;8:28. doi: 10.1186/s13068-015-0212-4 (PMC4355990; doi:10.1186/s13068-015-0212-4)
Supplement: Additional file 1: — Supporting material. Figure S1. Growth curves of non-diatom species that were considered in this study. See Experimental section for culture conditions. Figure S2. Growth curves of non-diatom species that were considered in this study. See Experimental section for culture conditions. Figure S3. Proton NMR spectra of extracts of T. weissflogii under Si-limited (a), N-limited (b), and replete (c) conditions. Figure S4. Proton NMR spectra of extracts of C. cryptica under Si-limited (a), N-limited (b), and replete (c) conditions. Table S1. Cell size of microalgae considered in this study. Table S2. Fatty acid composition of TAG in T. weissflogii and C. cryptica under nutrient limitation. [file 13068_2015_212_MOESM1_ESM.doc]

**SUPPORTING MATERIAL**

# Potential of lipid metabolism in marine diatoms for biofuel production

Giuliana d’Ippolito,* Angela Sardo, Debora Paris, Filomena Monica Vella, Maria Grazia Adelfi, Pierpaolo Botte, Carmela Gallo and Angelo Fontana

**Figure S1**. Growth curves of non-diatom species that were considered in this study. See Experimental Section for culture conditions.

**
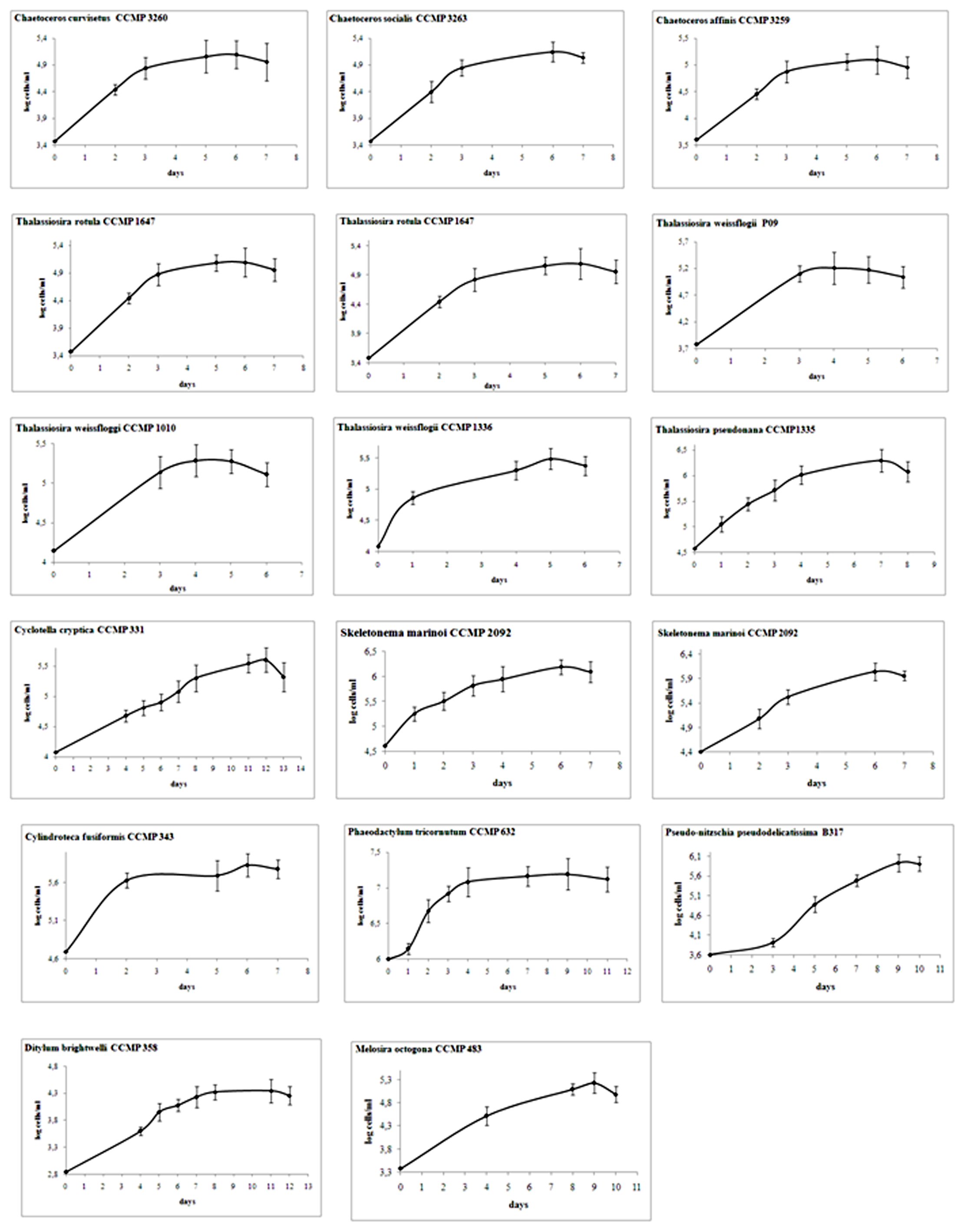
**

**Figure S2**. Growth curves of non-diatom species that were considered in this study. See Experimental Section for culture conditions.


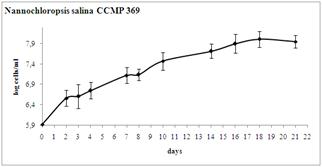


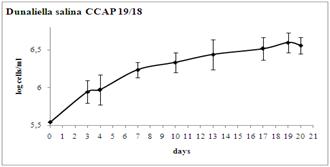


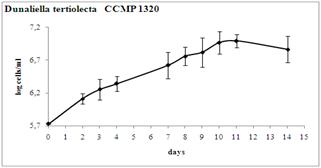


**Figure S3**. Proton NMR spectra of extracts of *T. weissflogii* under Si-limited (a), N-limited (b) and replete (c) conditions.


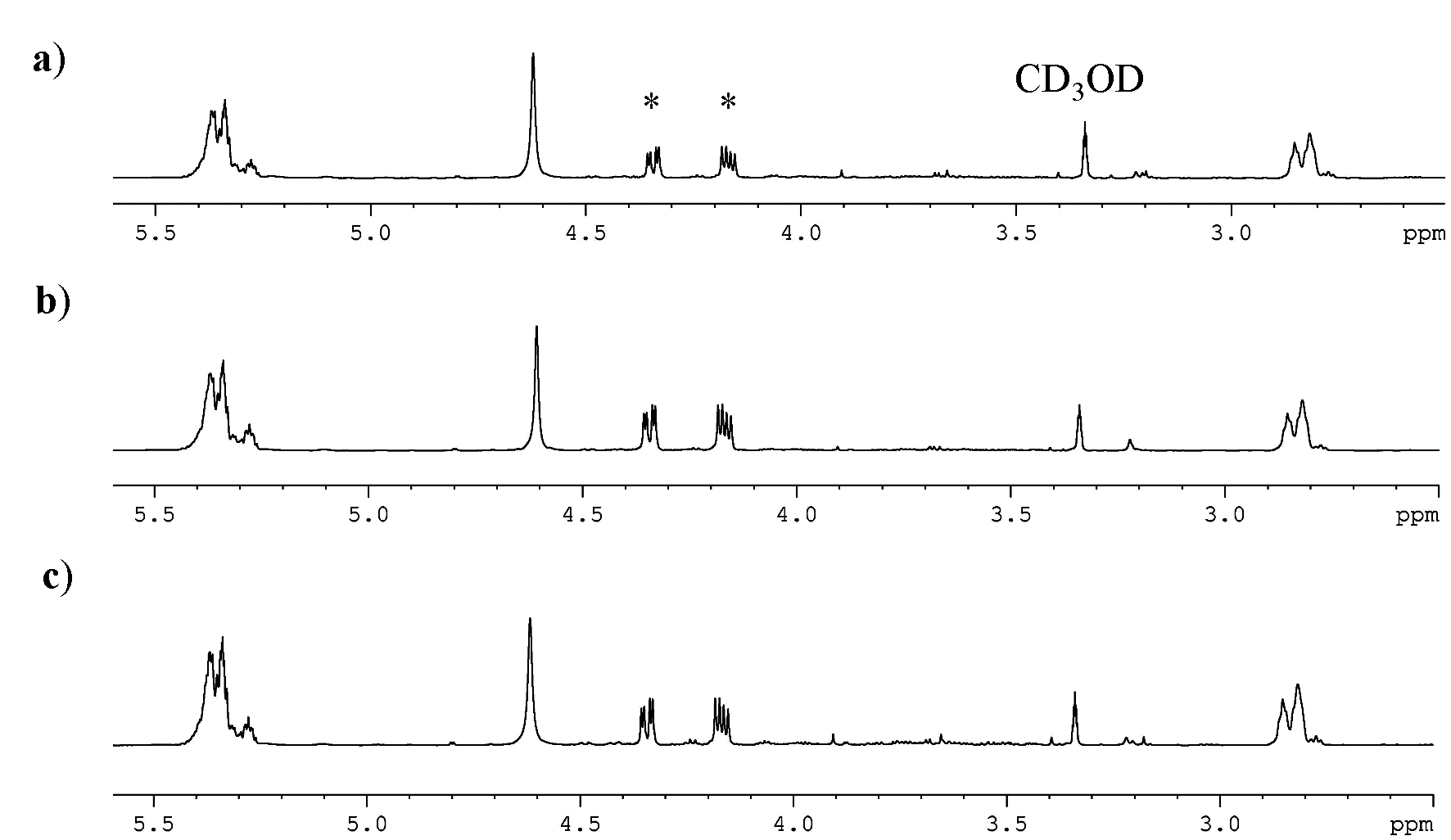


Symbols indicate the signals of TAGs that are used for quantitative analysis according to Ref. 41.

**Figure S4**. Proton NMR spectra of extracts of *C. cryptica* under Si-limited (a), N-limited (b) and replete (c) conditions.


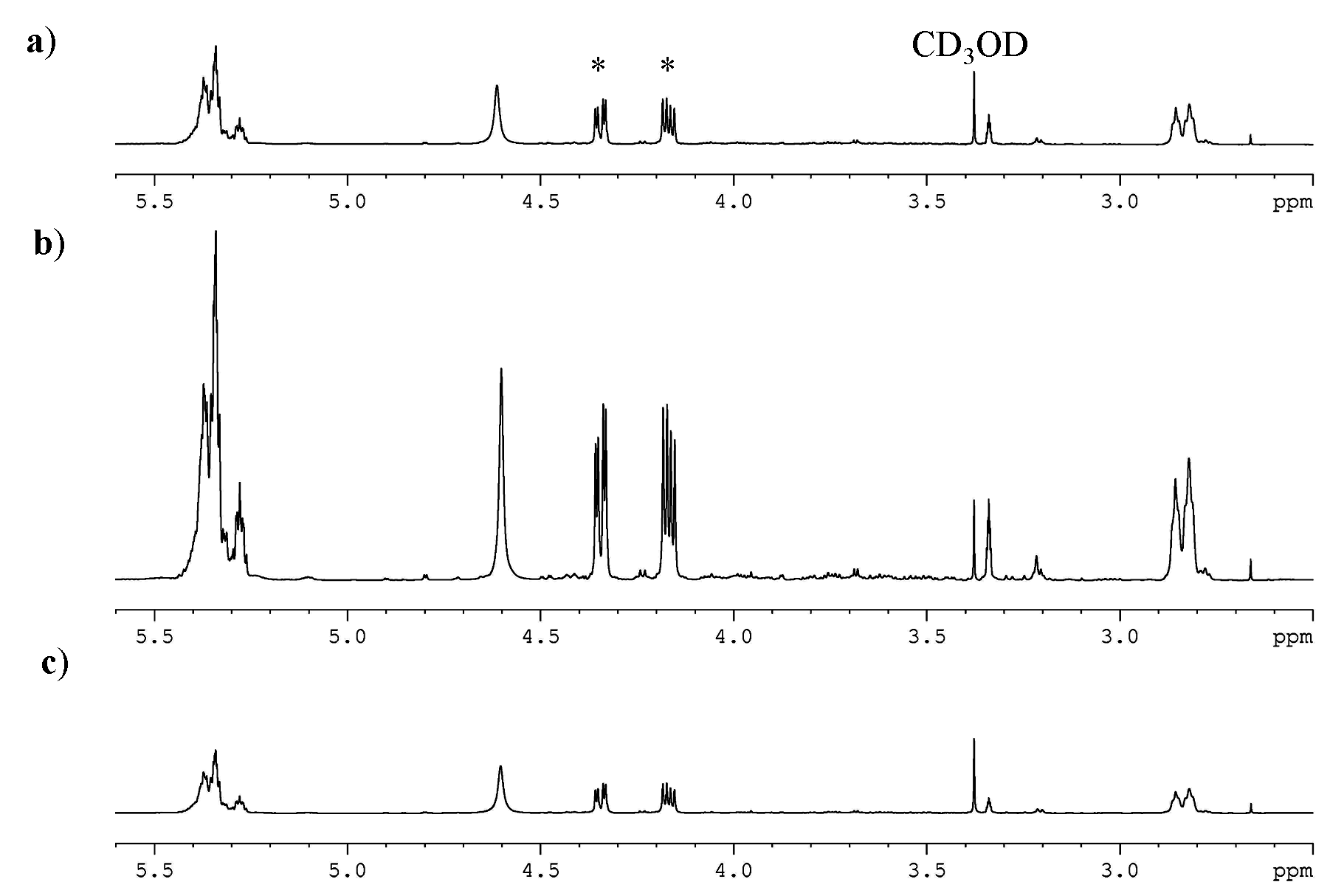


Symbols indicate the signals of TAGs that are used for quantitative analysis according to Ref. 41.

**Table S1**. Cell size of microalgae considered in this study.

| **Microalgae** | **Size (l=lenght; w=width)** |
| --- | --- |
| *Chaetoceros curvisetus CCMP 3260* | l=22-36 µm w=8-10 µm |
| *Chaetoceros socialis CCMP 3263* | l=6-12 µm w=4-8 µm |
| *Chaetoceros affinis CCMP 3259* | l=18-34 µm w=6-24µm |
| *Thalassiosira rotula CCMP 1647* | l=18-30 µm w=16-20 µm |
| *Thalassiosira rotula CCMP 3264* | l=16-24 µm w=16-19 µm |
| *Thalassiosira weissflogii P09* | l=12-20 µm w=10-12 µm |
| *Thalassiosira weissflogii CCMP 1010* | l=13-18 µm w=10-12 µm |
| *Thalassiosira weissflogii CCMP1336* | l=12-22 µm w=10-12 µm |
| *Thalassiosira pseudonana CCMP 1335* | l=4-6 µm w=4-5µm |
| *Cyclotella cryptica CCMP 331* | l=11-16 µm w=9-10 µm |
| *Skeletonema marinoi CCMP 2092* | l=4-6 µm w=4-5 µm |
| *Skeletonema marinoi CCMP 2052* | l=4-6 µm w=4-5 µm |
| *Cylindroteca fusiformis CCMP 343* | l=54-74 µm w=2-7 µm |
| *Phaeodactylum tricornutum CCMP 632* | l=18-26 µm w=2-3 µm |
| *Pseudo-nitzschia pseudodelicatissima B317* | l=54-87µm w= 1-1.6 µm |
| *Ditylum brightwelli CCMP 358* | l=40-81 µm w=12-38 µm |
| *Melosira octogona CCMP 483* | l=16-24 µm w=14-26 µm |
| *Nannochloropsis salina CCMP 369* | l=3-8 µm w=2-4 µm |
| *Dunaliella salina CCAP19/18* | l=6-9 µm w=0-2 µm |
| *Dunaliella tertiolecta CCMP 1320* | l=6-9 µm w=0-2 µm |
| *Chlamydomonas sp. CCMP 222* | l=7-14 µm w=5-10 µm |

**Table S2**. Fatty acid composition of TAG in *T. weissflogii* and *C. cryptica* under nutrient limitation.
